# Impact of the cone operation on left ventricular size, function, and dyssynchrony in Ebstein anomaly: a cardiovascular magnetic resonance study

**DOI:** 10.1186/s12968-018-0452-0

**Published:** 2018-05-21

**Authors:** Rebecca S. Beroukhim, Linyuan Jing, David M. Harrild, Brandon K. Fornwalt, Abba Mejia-Spiegeler, Jonathan Rhodes, Sitaram Emani, Andrew J. Powell

**Affiliations:** 1 Department of Pediatrics, Massachusetts General Hospital, 175 Cambridge Street, 5th Floor, Boston, MA 02114 USA; 2 Department of Cardiology, Boston Children’s Hospital, Boston, MA USA; 3 Department of Pediatrics, Harvard Medical School, Boston, MA USA; 4 Department of Imaging Science and Innovation, Geisinger, Danville, PA USA; 5 Department of Radiology, Geisinger, Danville, PA USA; 6 Department of Cardiac Surgery, Boston Children’s Hospital, Boston, MA USA; 7 Department of Surgery, Harvard Medical School, Boston, MA USA

**Keywords:** Ebstein anomaly, Cardiovascular magnetic resonance, Strain, Dyssynchrony, Cone operation

## Abstract

**Background:**

In addition to tricuspid regurgitation (TR) and right ventricular (RV) enlargement, patients with Ebstein anomaly are at risk for left ventricular (LV) dysfunction and dyssynchrony. We studied the impact of the cone tricuspid valve reconstruction operation on LV size, function, and dyssynchrony.

**Methods:**

All Ebstein anomaly patients who had both pre- and postoperative cardiovascular magnetic resonance (CMR) studies were retrospectively identified. From cine images, RV and LV volumes and ejection fractions (EF) were calculated, and LV circumferential and longitudinal strain were measured by feature tracking. To quantify LV dyssynchrony, temporal offsets (TOs) were computed among segmental circumferential strain versus time curves using cross-correlation analysis and patient-specific reference curves. An LV dyssynchrony index was calculated as the standard deviation of the TOs.

**Results:**

Twenty patients (65% female) were included with a median age at cone operation of 16 years, and a median time between pre- and postoperative CMR of 2.8 years. Postoperatively, there was a decline in the TR fraction (56 ± 19% vs. 5 ± 4%, *p* < 0.001), RV end-diastolic volume (EDV) (242 ± 110 ml/m^2^ vs. 137 ± 82 ml/m^2^, *p* < 0.001), and RV stroke volume (SV) (101 ± 35 vs. 51 ± 7 ml/m^2^, *p* < 0.001). RV EF was unchanged. Conversely, there was an increase in both LV EDV (68 ± 13 vs. 85 ± 13 ml/m^2^, *p* < 0.001) and LV stroke volume (37 ± 8 vs. 48 ± 6 ml/m^2^, *p* < 0.001). There was no change in LV EF, or global circumferential and longitudinal strain but basal septal circumferential strain improved (16 ± 7% vs. 22 ± 5%, *p* = 0.04). LV contraction become more synchronous (dyssynchrony index: 32 ± 17 vs. 21 ± 9 msec, *p* = 0.02), and the extent correlated with the reduction in RV EDV and TR.

**Conclusions:**

In patients with the Ebstein anomaly, the cone operation led to reduced TR and RV stroke volume, increased LV stroke volume, improved LV basal septal strain, and improved LV synchrony. Our data demonstrates that the detrimental effect of the RV on LV function can be mitigated by the cone operation.

## Background

Ebstein anomaly of the tricuspid valve is characterized by a failure of delamination of the septal and posterior leaflets, redundancy and fenestrations of the anterior leaflet, and dilatation of the annulus. This combination of factors often leads to progressive tricuspid regurgitation (TR), and right ventricular (RV) enlargement and dysfunction. Although the most evident pathologic processes affect the RV, the left ventricle (LV) is also abnormal. Paradoxical septal motion, LV dysfunction, LV dyssynchrony, and LV patchy microfocal interstitial fibrosis have all been reported in patients with Ebstein anomaly [1–3].

The cone operation is a surgical technique aimed at reducing the severity of TR in patients with Ebstein anomaly. The tricuspid valve is reconstructed to form a bileaflet valve with hingepoints at the normal annular position, and the atrialized portion of the RV is plicated [4]. Studies on the short- and medium-term outcomes of the cone operation have reported a dramatic reduction in TR and RV end-diastolic volume (EDV) [2, 5, 6]; however, the impact on LV mechanics and synchrony have not been fully described. Whereas paradoxical septal motion in RV volume loading lesions has been shown to decrease LV EDV [7, 8], the opposite effect might be expected after surgical treatment of TR [9]. We, therefore, aimed to measure the impact of the cone operation on LV volume, strain, and dyssynchrony. A deeper understanding of these changes should provide insight into the pathophysiology of Ebstein anomaly as well as the efficacy of the cone operation. We chose cardiovascular magnetic resonance (CMR) as our principle assessment tool because of its superior ability to visualize all LV segments pre- and postoperatively.

## Methods

### Subjects

A database search at Boston Children’s Hospital identified all patients with Ebstein anomaly who had undergone CMR both before and after a cone operation. Patients were excluded from the study cohort if they had a superior cavopulmonary anastomosis at the time of either CMR, pulmonary valve stenosis, a history of a prior tricuspid valvuloplasty, a history of a myocardial infarction, or poor quality CMR data. The Boston Children’s Hospital Committee on Clinical Investigation granted permission for this study and waived the requirement for informed consent.

### CMR

CMR studies were performed on a 1.5 Tesla CMR scanner (Achieva, Philips Medical Systems, Best, the Netherlands). Electrocardiogram (ECG) gated balanced steady-state free precession cine images in ventricular two-chamber, four-chamber, and short-axis planes were acquired during 8–12 s breath-holds. Short-axis images were acquired as a stack of contiguous slices to completely encompass both ventricles. Acquisition parameters included the following: repetition time 3.4 msec, echo time 1.7 msec, flip angle 60°, in-plane resolution 1.5–1.7 mm, slice thickness 6–8 mm, and 20 phases per cardiac cycle reconstructed to 30 phases per cycle.

### Ventricular volumes and tricuspid regurgitation

Biventricular volumes and mass were calculated using a standard summation of disks methodology. Epicardial and endocardial boundaries at the end-diastolic frame, and endocardial boundaries at the end-systolic frame were manually traced on the cine short-axis image stack using commercial software (QMass, Medis, Leiden, the Netherlands). The short-axis images were cross-referenced with the 2- and 4-chamber views to facilitate boundary identification. Ventricular volume measurements were performed by a single unblinded observer (RSB, 10 years of CMR experience).The RV volumes reported in the results included the atrialized portion of the RV as this was thought to best demonstrate volume changes that were related to volume load reduction, given that the cone operation repositions the functional annulus. For TR fraction calculation, RV EDV and end-systolic volume were measured excluding the atrialized portion of the RV as this provides a more accurate calculation of RV stroke volume (SV). TR fraction was calculated as ((RV SV – LV SV) ÷ RV SV) in the absence of a shunt and other valve regurgitation, or as ((RV SV – main pulmonary artery antegrade flow) ÷ RV SV).

### Strain

LV circumferential and longitudinal strain were measured on cine images using a displacement-based custom feature tracking algorithm written in Matlab (Mathworks, Natick, Massachusetts, USA). A detailed description of the algorithm, including demonstration of good inter-test reproducibility, has been reported [10]. Strain analysis was performed by a single blinded observer (AM, 1 year of experience) with confirmation by another blinded observer (LJ, 5 years of experience). For LV circumferential strain, all short-axis slices located between the apex and the mitral valve plane at end-systole were analyzed. LV endocardial borders throughout the cardiac cycle were semi-automatically determined using a level-set algorithm. For each slice, 12 evenly spaced nodes around the endocardial boundary were defined on the first frame and tracked throughout the cardiac cycle to generate circumferential strain versus time curves for the 12 segments. As a result, a total of 72–96 segmental strain versus time curves (6–8 slices) covering the entire LV were generated for each patient. A global circumferential strain versus time curve was computed by averaging all segmental strain values in each frame, and the most negative value of the global strain curve versus time curve was reported as peak global circumferential strain. Similarly, for longitudinal strain, the LV endocardial borders of the 2-chamber and 4-chamber slices were identified, and segmental strain versus time curves were averaged to calculate peak global longitudinal strain. To quantify regional circumferential strains, the LV short-axis images were systematically divided into 9 regions: septal, anterior, and inferior regions at the basal, mid, and apical levels. Segmental circumferential strain versus time curves within each region were averaged to compute regional peak circumferential strains. For simplicity, the absolute values of the peak strains are reported in the results.

### Dyssynchrony

LV dyssynchrony was assessed by analyzing the segmental circumferential strain versus time curves. For each CMR study, a reference curve was derived from the 72–96 segmental curves covering the entire LV using a QT-clustering algorithm [10, 11]. Then, for each segmental curve, the temporal offset (TO) relative to the reference curve was calculated using cross-correlation analysis. The TO is the time displacement needed to maximize the overlap of the two curves and has been used to provide more robust results compared to a simple analysis of the time to peak strain [10, 11]. The LV dyssynchrony index was defined as the standard deviation of all the TOs so that a higher index indicates greater dyssynchrony [10]. The dyssynchrony index was divided by the square root of the R-R interval measured in seconds to account for heart rate variability. To facilitate analysis, segments were grouped and assigned to an 18-segment circumferential polar plot, and the mean TO for each segment was overlaid on the plot. The QRS duration was obtained from pre- and postoperative electrocardiograms.

### Cardiopulmonary exercise testing

Exercise test data from the subset of patients who had cardiopulmonary exercise testing (CPET) both before and after the cone operation were reviewed. Only studies in which the patient expended an adequate effort (peak respiratory exchange ratio > 1.09 or peak heart rate > 85% predicted) were included in the analysis. CPET studies were performed using a CardiO2 metabolic cart (Medical Graphics Corporation, St. Paul, Minnesota, USA). Peak oxygen consumption (VO_2_), oxygen pulse at peak exercise (expressed as percent predicted for age, gender, and body size), and VE/VCO_2_ slope (an index of the efficiency of gas exchange during exercise) were abstracted from the clinical reports.

### Statistics

Demographic, clinical, and CMR results are expressed as mean ± standard deviation, or median and range, as appropriate. Pre- and postoperative values that were normally distributed according to the Shapiro-Wilk test and graphical inspection were compared using a two-tailed Student’s paired t-test. Two ventricular and two strain measures were non-normally distributed and their pre-post change was assessed using the Wilcoxon signed rank test. The mean percentage change in RV EDV and TR in patients with a negative versus positive change in LV dyssynchrony index was compared using a two-sided Student’s t-test. The association between the change in LV dyssynchrony index versus the percentage change in RV EDV and TR was assessed by linear regression modeling. *P*-values < 0.05 were considered statistically significant. Analyses were performed using SAS software (version 9.4, SAS Institute, Inc., Cary, North Carolina, USA).

## Results

### Subjects

Of the 38 patients who had undergone the cone operation and received both pre- and postoperative CMR examinations, 18 were excluded (prior tricuspid valvuloplasty, *n* = 7; inadequate image quality, *n* = 6; bidirectional superior cavopulmonary anastomosis, *n* = 2; pulmonary stenosis, *n* = 2; and LV infarct, *n* = 1). The remaining 20 patients formed the study group, and their demographic and clinical data are shown in Table 1. The cone operations were performed between 2006 and 2014, and the median age at surgery was 16 years (range, 5–41 years). The timing of surgery was determined by the primary cardiologist and surgeon, and was largely based on symptoms, and degree of TR and RV dilation. The indications included severe TR and RV enlargement (*n* = 17), exercise intolerance (*n* = 9), atrial arrhythmia (*n* = 3), worsening fatigue (*n* = 2), desaturation with exercise (*n* = 2), and progressive cyanosis at rest (*n* = 1). Concurrent procedures included the following: patent foramen ovale closure (*n* = 12), tricuspid annuloplasty ring (*n* = 9), right atrial CryoMaze (*n* = 8), and accessory pathway cryoablation (*n* = 1). Postoperatively, one patient developed dehiscence of the annular plication and underwent reoperation with an annuloplasty ring placement 4 months after initial cone operation. Another patient returned to the operating room for residual TR 4 days after the initial operation. Postoperative CMR studies were performed following these reoperations. The median time between pre- and postoperative CMR examinations was 2.8 years (range 0.3–7.2 years). All patients were in normal sinus rhythm at the CMR examinations. The heart rate and blood pressure were unchanged between the pre- and postoperative CMR examinations, and the QRS duration increased (Table 1).

**Table 1:** Pre- and postoperative demographic and clinical data (*N* = 20)

	Preoperative	Postoperative	*P*-value
Age at CMR (years)	16 (5–41)	19 (8–42)	< 0.001
Body surface area (m^2^)	1.5 ± 0.4	1.7 ± 0.3	0.002
Systolic blood pressure (mm Hg)	113 ± 17	116 ± 11	0.39
Diastolic blood pressure (mm Hg)	71 ± 15	68 ± 8	0.31
Heart rate (bpm)	86 ± 13	84 ± 17	0.56
QRS duration (msec)	115 ± 24	137 ± 24	< 0.001

Values are median (range) or mean ± standard deviation. CMR = cardiovascular magnetic resonance

### Ventricular volumes, mass, and ejection fraction

Pre- and postoperative CMR parameters are shown in Table 2. For the RV, there was a marked reduction in TR, end-diastolic volume index (EDVi), end-systolic volume index (ESVi), and stroke volume index (SVi). For the LV, there was a significant increase in the EDVi, ESVi, and SVi. RV and LV ejection fractions (EF) were unchanged.

**Table 2:** Pre- and postoperative CMR parameters (*N* = 20)

	Preoperative	Postoperative	*P*-value
TR fraction (%)	56 ± 19	5 ± 4	< 0.001
Right ventricle			
End-diastolic volume (ml/m^2^)	242 ± 110	137 ± 82	< 0.001
End-systolic volume (ml/m^2^)	141 ± 80	86 ± 65	0.001
Stroke volume (ml/m^2^)	101 ± 35	51 ± 7	< 0.001
Mass (g/m^2^)	36 ± 15	29 ± 12	0.002
Ejection fraction (%)	43 ± 9	41 ± 9	0.13
Left ventricle			
End-diastolic volume (ml/m^2^)	68 ± 13	85 ± 13	< 0.001
End-systolic volume (ml/m^2^)	31 ± 8	37 ± 10	0.001
Stroke volume (ml/m^2^)	37 ± 8	48 ± 6	< 0.001
Mass (g/m^2^)	45 ± 10	52 ± 8	< 0.001
Ejection fraction (%)	55 ± 7	57 ± 6	0.35

Values are mean ± standard deviation. TR = tricuspid regurgitation

### Left ventricular strain

**Fig. 1:**
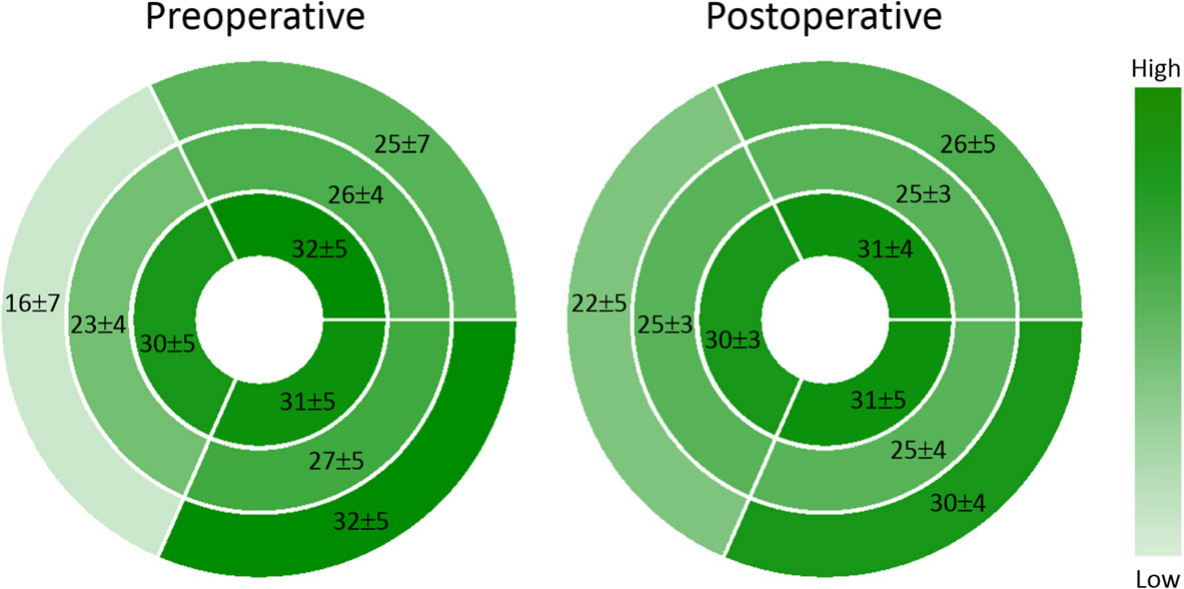
Circumferential polar plots demonstrating left ventricular pre- and postoperative regional circumferential strain (%, mean ± standard deviation). There was no significant change in strain for any segment except for the basal septum (16±7% vs. 22±5%, *p* = 0.03)

LV strain data are shown in Table 3 and Fig. 1. LV global circumferential and longitudinal strain did not change between the pre- and postoperative CMR examinations. Regarding regional function, preoperative basal septal strain was diminished compared to other segments (basal septal strain 16 ± 7% vs. global circumferential strain 26 ± 4%, *p* < 0.001). Although the basal septal segment showed improved strain postoperatively (pre: 16±7% vs. post: 22±5%, *p* = 0.002), it remained lower than the other segments (Fig. 1).

**Table 3:** Preoperative and postoperative left ventricular strain and dyssynchrony index (*N* = 20)

	Preoperative	Postoperative	*P*-value
Global longitudinal strain (%)	20 ± 3	19 ± 4	0.12
Circumferential strain (%)			
Global	26 ± 4	27 ± 3	0.45
Septum	22 ± 5	25 ± 3	0.03
Anterior	27 ± 4	27 ± 3	0.95
Inferior	29 ± 4	29 ± 4	0.62
Dyssynchrony index (msec)	32 ± 17	21 ± 9	0.02
Dyssynchrony index (msec) ÷ √RR (sec)	38 ± 20	23 ± 10	0.01

Values are mean ± standard deviation

### Left ventricular dyssynchrony

**Fig. 2:**
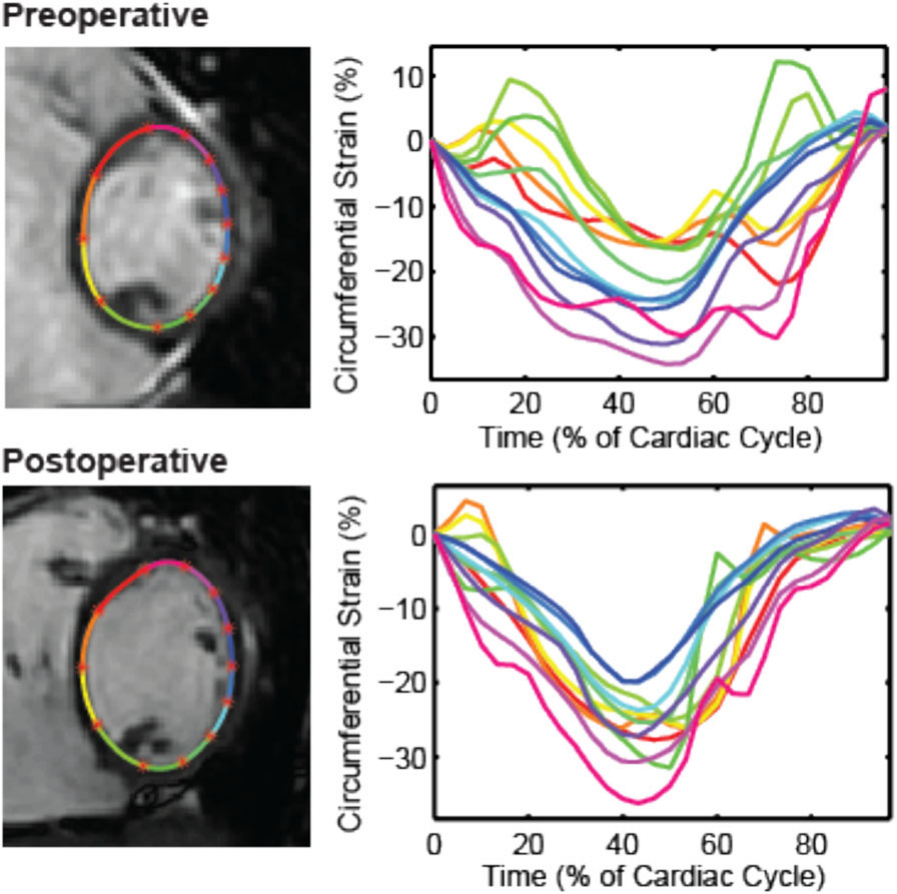
Representative patient demonstrating a change in the dyssynchrony index between pre- and postoperative CMR examinations. The images show segmental strain versus time curves at the mid-ventricular level. In this patient, the dyssynchrony index decreased from 57 to 13 msec

**Fig. 3:**
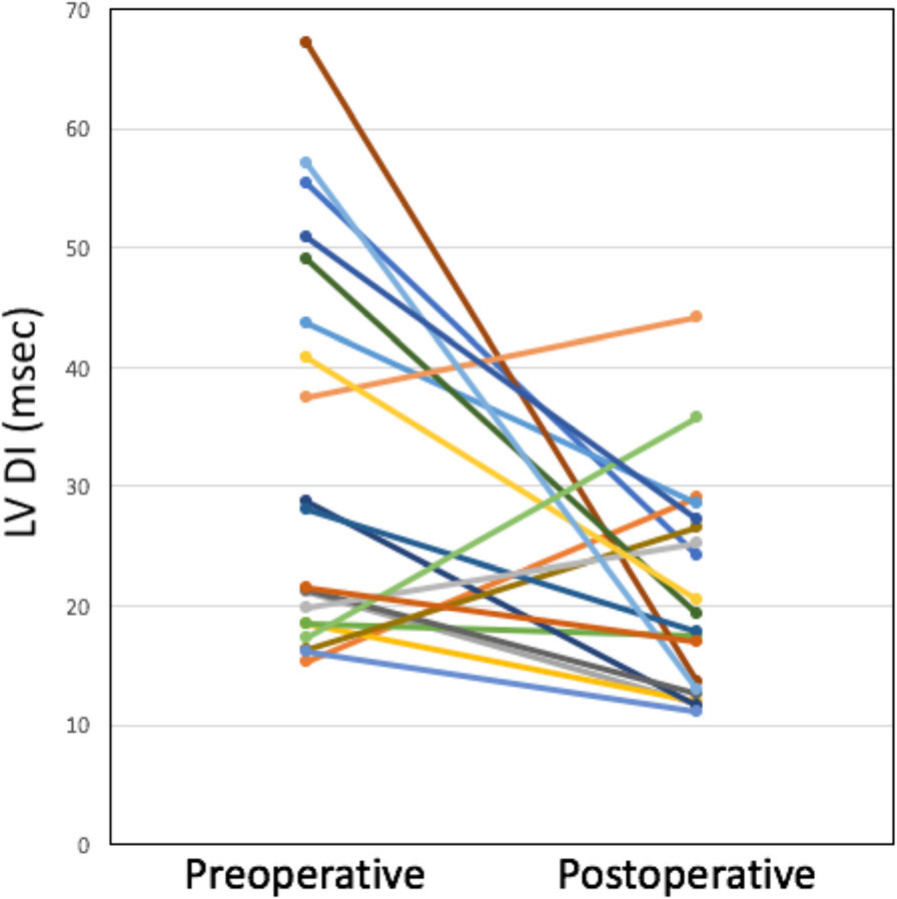
Pre- and postoperative LV dyssynchrony index for each of the 20 patients

As shown in Table 3, the LV dyssynchrony indeximproved between the pre- and postoperative CMR studies, both when assessed as an absolute value and when divided by the square root of the R-R interval. Data from a representative patient are shown in Fig. 2. The extent of improvement in the LV DI was associated with a greater decrease in both RV EDVi (*p* = 0.01) and TR fraction (*p* = 0.022). There were 5 patients in whom the LV dyssynchrony index did not improve (Fig. 3). For these 5 patients, the percent decrease in RV EDVi was less compared to the other 15 patients (16 ± 15% versus 48 ± 13%, *p* < 0.001).

**Fig. 4:**
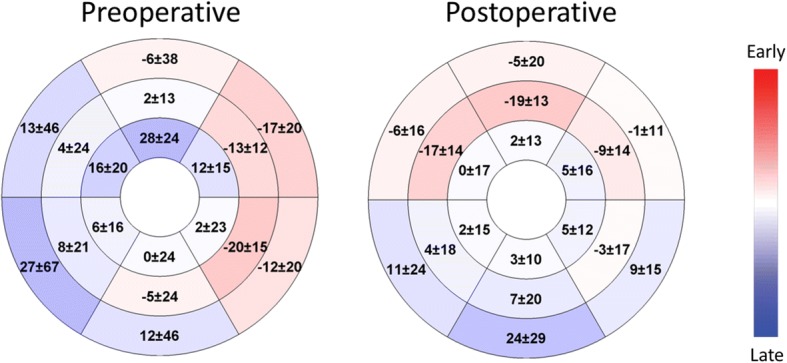
Circumferential polar plot of the mean temporal offset (msec) for each left ventricular segment at the preoperative and postoperative CMR examinations. Negative numbers represent early contraction (red) and positive numbers represent late contraction (blue)

Figure. 4 shows the mean TO among all patients for each segment of an 18-segment model pre- and postoperatively; in other words, using segmental circumferential strain data from all patients, it shows the temporal pattern of contraction before and after the cone operation. Preoperatively, on average, the free wall contracted before the septum. Postoperatively, the TOs were smaller and had a narrower range.

A higher preoperative LV dyssynchrony indexwas correlated with a higher preoperative RV EDVi (*R* = 0.66, *p* < 0.01) and a higher postoperative LV dyssynchrony indexwas correlated with a higher postoperative RV EDVi (*R* = 0.46, *p* = 0.04). LV dyssynchrony indexwas not significantly correlated with TR fraction.

### Cardiopulmonary exercise testing

Ten patients underwent both pre- and postoperative CPET: 7 paired studies using an upright bicycle ergometer and 3 paired studies using the Bruce treadmill protocol. The median interval between studies was 1.4 yrs. (range, 0.5–2.9 yrs). No significant change in peak VO2, oxygen pulse at peak exercise, or VE/VCO_2_ was observed (Table 4). The changes in TR fraction, RV and LV volumes, septal circumferential strain, and dyssynchrony reported above in the whole cohort remained significant in this subgroup.

**Table 4:** Pre- and postoperative cardiopulmonary exercise test data (*N* = 10)

	Preoperative	Postoperative	*P*-value
Age (years)	16 (7–42)	17 (9–42)	< 0.001
BMI (kg/m^2^)	21 ± 5	21 ± 9	0.87
Maximum oxygen consumption (% predicted)	77 ± 16	81 ± 14	0.44
Oxygen pulse (% predicted)	84 ± 17	87 ± 23	0.67
VE/VCO_2_ slope	30 ± 5	28 ± 4	0.35
RER	1.2 ± 0.1	1.2 ± 0.1	0.09
Peak heart rate (% predicted)	92 ± 10	89 ± 12	0.47

Values are median (range) or mean ± standard deviation. BMI = body mass index; VE/VCO2 = minute ventilation/carbon dioxide production; RER = respiratory exchange ratio

## Discussion

In this retrospective study of 20 patients with Ebstein anomaly, we utilized CMR to assess the impact of the cone operation on ventricular volumes, LV strain, and LV dyssynchrony. In conjunction with a marked reduction in TR, there was a *decrease* in RV EDVi, ESVi, and SVi, and no change in EF. For the LV, there was an *increase* in EDVi, ESVi, and SVi, and no change in EF. LV global circumferential and longitudinal strain were unchanged whereas basal septal strain was augmented. LV dyssynchrony index improved and the extent correlated with the reduction in RV EDVi and TR. The delayed septal contraction seen preoperatively was less pronounced postoperatively.

### Prior studies

CMR is an effective tool to study the impact of the cone operation for Ebstein anomaly because of its ability to clearly visualize all segments of both ventricles and to quantify tricuspid regurgitation. Our study is the largest, most comprehensive report thus far using CMR, and the only one to investigate LV synchrony. As in our work, prior studies have found that the cone operation leads to a decrease in RV EDV [6, 12–14]. The effect on RV EF is less consistent with some investigations showing no change [6, 12] as we found, and others showing a decrease [13, 14]. This discrepancy may reflect variations among the studies in the indications for surgery and timing of the postoperative evaluation, and requires further study. Our finding of an increase in LV EDV and no change in LV EF following the cone operation is in agreement with prior studies [12, 13]. The only prior work to investigate the impact on synchrony examined the RV. Li et al. reported that the cone operation led to more uniform movement of the tricuspid annulus [6]. For our study, we chose not to measure RV synchrony because feature tracking of the RV is less robust, and comparison between segments pre- and postoperatively would have been confounded by the RV plication which is done as part of the cone operation.

### Physiology and implications

The reconstruction of the TV with the cone operation led to a reduction of TR and RV volume load, and a decrease in the RV EDV. The reduction in RV EDV likely contributed to the increase LV EDV through ventricular-ventricular interaction. Lower volume and likely filling pressure in the RV may alter the septal configuration and transmural pressure leading to improved LV filling. A similar increase in LV EDV is seen with other procedures that relieve RV volume load such as atrial septal defect closure [9] or percutaneous pulmonary valve replacement [15].

Clinicians have noted abnormal septal movement in patients with Ebstein anomaly but the mechanism has not been fully elucidated. Goleski et al. used mathematical models to describe the apparent septal dyskinesis in both pre- and postoperative patients with Ebstein anomaly, and concluded that this phenomenon is more likely related to cardiac translation than a true abnormality of septal movement [1]. However, our data demonstrating an improvement in basal septal strain following the cone operation suggests that the septal abnormality is mostly confined to the basal septum, and likely due to a direct mechanical impact of the atrialized RV on the LV. Furthermore, our observation that the cone operation leads to an earlier contraction of the basal septum also suggests that the septum is particularly vulnerable to the abnormal mechanical movement and pressure changes in the adjacent atrialized RV. Despite the improvement seen in LV dyssynchrony, the QRS duration increased. This increase may have been related to surgical manipulation of the RV leading to slower intraventricular conduction.

On average, the cone operation was associated with a 25% increase in the LV EDV. As heart rate and ejection fraction were unchanged, this led to a similar increase in cardiac output. Despite this, we found no significant improvement in either peak VO2 or oxygen pulse on CPET. There are several possible explanations for a lack of demonstrable increase in exercise capacity. Pre- and postoperative CPET data were only available in half of our study cohort thus limiting the ability to detect small changes. The CMR data were obtained at rest and may not reflect the physiology during exercise. Lastly, peak VO_2_ is a measure of both cardiac performance and musculoskeletal conditioning. Our patients may have been adversely affected by postoperative deconditioning, which is a phenomenon commonly encountered in patients undergoing even less extensive congenital heart disease surgery [16].

By demonstrating an improvement in LV SV and synchrony with the cone operation, our study illustrates that the detrimental effects of Ebstein anomaly and TR are not simply confined to the right heart but also extend to the left heart. As such, they provide an additional rationale for performing the operation beyond preservation of right heart function and lowering the risk of atrial arrhythmia. This “LV indication” is further supported by reports showing that LV dyssynchrony is associated with worse heart failure class and higher brain natriuretic peptide levels in Ebstein patients [3] and ventricular tachycardia and death in repaired tetralogy of Fallot patients [17]. Future studies should thus examine the relationship between post-operative improvement in LV functional parameters and long-term morbidity and mortality. Similarly, the effect of age at the cone operation on improvement in LV parameters should be investigated to better understand the optimal timing for intervention.

### Limitations

Because Ebstein anomaly is rare, our study was limited by a small sample size. Also, the retrospective study design led to a variable timing of the pre- and postoperative CMR studies. Strain analysis using feature tracking on 2D cine images is subject to some inherent limitations including insensitivity to through-plane motion effects. Newer methods such as three-dimensional displacement encoding with stimulated echoes (DENSE) can overcome such limitations, but are not yet widely available [18]. Also, the temporal resolution of the cine imaging was relatively low; consequently, small changes in synchrony parameters may have gone undetected. However, as the temporal resolution for each patient was similar on the pre- and postoperative studies, the synchrony changes which were identified are likely valid. TR fraction calculations were derived from RV volume measurements which may be particularly challenging at the ventricular base in Ebstein patients. Newer 4D flow measurement techniques may allow for direct quantification of the tricuspid regurgitation volume, and improved accuracy [19]. The effect of preoperative LV interstitial fibrosis on postoperative LV strain and dyssynchrony was not assessed.

## Conclusion

In patients with the Ebstein anomaly, the cone operation leads to a reduction in TR and RV volume, an increase in LV volume/stroke volume, and improved LV synchrony. Future studies should address whether changes in these parameters relate to long-term outcome.

## Abbreviations

CMR: Cardiovascular magnetic resonance
CPET: Cardiopulmonary exercise test
ECG: Electrocardiogram
EDV: End-diastolic volume
EDVi: End-diastolic volume index
EF: Ejection fraction
ESV: end-systolic volume
LV: Left ventricle/left ventricular
RER: Respiratory exchange ratio
SV: Stroke volume
SVi: Stroke volume index
TO: temporal offset
TR: Tricuspid regurgitation
VO_2_: Peak oxygen consumption
